# Fluorescence optical imaging for treatment monitoring in patients with early and active rheumatoid arthritis in a 1-year follow-up period

**DOI:** 10.1186/s13075-019-1989-5

**Published:** 2019-09-18

**Authors:** Anne-Marie Glimm, Lisa Ines Sprenger, Ida Kristin Haugen, Ulrich Mansmann, Sandra Hermann, Thomas Häupl, Paula Hoff, Gerd-Rüdiger Burmester, Marina Backhaus, Lien Le, Sarah Ohrndorf

**Affiliations:** 1 Department of Rheumatology and Clinical Immunology, Charité-Universitätsmedizin Berlin, Charitéplatz 1, 10117 Berlin, Germany; 2 Department of Rheumatology, Diakonhjemmet Hospital, Oslo, Norway; 3 Institut für medizinische Informationsverarbeitung, Biometrie und Epidemiologie (IBE), Ludwig-Maximilians-Universität München, Munich, Germany; 4 Innere Medizin – Bereich Rheumatologie und Klinische Immunologie, Park-Klinik Weißensee, Berlin, Germany

**Keywords:** Arthritis, rheumatoid, Fluorescence, Ultrasonography, Follow-up studies

## Abstract

**Background:**

Fluorescence optical imaging (FOI) enables visualization of inflammation in the hands in rheumatic joint diseases with currently a lack of long-term follow-up studies.

**Objective:**

To investigate FOI for treatment monitoring in a homogenous cohort of patients with early (disease duration < 2 years) and active (DAS28 > 3.2) RA over a period of 12 months.

**Methods:**

Thirty-five RA patients (24 (68.6%) females, mean age 53.3 years (SD 13.6)) were investigated clinically by DAS28, tender joint count (TJC) and swollen joint count (SJC) and by FOI in phases 1–3 and PrimaVistaMode (PVM) before therapy change and after 12 months. The FOI activity score (FOIAS) was calculated based on individual joint scores from 0 to 3 in 30 joints per patient, adding up to a sum score (0–90).

**Results:**

We found a statistically significant reduction of FOIAS in phase 1 from baseline (median 5.0, IQR 24.96) to follow-up (median 1.0, IQR 4.0) in all patients (*p* = 0.0045), both in responders and non-responders according to EULAR response criteria by DAS28. Statistically significant reductions over 12 months were found for median DAS28(ESR) 5.61 to 3.31, TJC 7.0 to 1.0, and SJC 5.0 to 1.0 (each *p* <  0.001). No statistically significant correlations were detected between the FOIAS change in phase 1 and DAS28(ESR), TJC, or SJC. Correlations between the other phases and clinical outcomes were weak to moderate.

**Conclusion:**

Reduced early enhancement in FOI phase 1 can be observed in clinically responding and non-responding early RA patients under treatment. Regarding potential marker performance, FOI probably shows a reduction of inflammation more objectively.

## Background

To monitor therapeutic response in patients with rheumatoid arthritis (RA), clinical disease activity scores such as DAS28 are applied [1]. Besides, more sensitive and objective imaging modalities are recommended in the clinical management of RA [2]. Magnetic resonance imaging (MRI) and musculoskeletal ultrasound (US) are both widely used in clinical practice and research within the field of RA [3–8]. MRI-detected pathologies such as synovitis and tenosynovitis are highly responsive to antirheumatic treatment [9–11]. However, MRI has the disadvantage of high costs, time exposure, and occasional contraindications (e.g., pacemaker and claustrophobia) [12]. In several studies, US-detected synovitis and tenosynovitis have also been shown to be sensitive to change under therapy, especially in Power Doppler mode (PDUS) [13–16] reflecting disease activity. US is a cost-effective, widespread method that is risk-free for patients, is indefinitely repeatable, and involves less inconvenience than MRI. Drawbacks may be the dependency on the examiner [17] and the inability to pairwise compare baseline and follow-up images immediately while investigating unless all images are saved for analysis later on; however, US images are usually saved as “still images” (lost of dynamic approach).

In search of an imaging method for the optimal detection of disease activity, new procedures are developed and investigated. Since 2009, the fluorescence optical imaging (FOI) “Xiralite” (Xiralite GmbH, Berlin, Germany) has been shown to detect inflammation in preclinical studies [18, 19] as well as in humans [20–26] in the joint regions of both hands. The basis of the Xiralite method is the demonstration of an impaired microcirculation caused by the inflammatory process of arthritis. Here, the enhancement of an intravenously applied dye indocyanine green (ICG) is evaluated. FOI is a non-ionizing technique that examines both hands in one session of 6 min. Besides, the examination itself can be performed by clinical assistants. Impediments in the sense of resulting contraindications are an impaired liver function, since the applied dye is primarily excreted biliarily [27]. Furthermore, an allergic reaction to the ICG solution can occur [28]. However, the overall risk of ICG to the patient is low [29].

Previous studies have demonstrated good agreements between FOI, clinical assessment, MRI, and US [21] as well as a moderate und substantial reliability for the scoring of FOI images [22]. Additionally, FOI may also detect subclinical inflammation [25]. Only one study has evaluated the responsiveness of FOI, so far. Meier et al. found a reduction in the signal intensity during therapy response in a group of patients with forms of different arthritis who were examined by a computer-based evaluation of FOI and MRI; however, the observed group was heterogeneous and only investigated over a time period of 6 months [30].

The aim of the present study was the investigation of FOI’s ability to reflect treatment response in a homogenous cohort of patients with early and active RA over a period of 12 months. Besides, we aimed for exploration of its correlations with clinical outcomes such as DAS28. The correlation with US as a common imaging modality in daily rheumatological practice was set as a secondary outcome.

## Methods

This study is a subproject (No. 7) of the Arthromark project as a national research network in Germany funded by the Federal Ministry of Education and Research (BMBF). The main goal of the several Arthromark subprojects is the identification of new biomarkers including the application and assessment of new and modern imaging techniques in terms of making a diagnosis and follow-up examinations in patients with RA, psoriasis arthritis, and spondyloarthritis [31].

In this subproject, we included 42 patients with early (disease duration < 24 months) and active (DAS28 > 3.2) RA, who started therapy with conventional synthetic disease-modifying antirheumatic drugs (csDMARDs) or escalated therapy with initiation of biologic therapy (bDMARD) after failure of conventional therapy. Over a period of 1 year, patients were examined clinically and by US (for further description of additional US, see Additional file 1) five times (baseline, after 6 weeks, and 3, 6, and 12 months). FOI was performed at baseline and after 12 months in 35 patients who were included in these analyses (in seven patients, the 12-month visit was not performed).

### Clinical and laboratory examination

A clinical assessment of tender (28 tender joint count (TJC)) and swollen joints (28 swollen joint count (SJC)) was performed. Patients self-reported their evaluation of the global disease activity and the current general joint pain (both on a visual analog scale (VAS) 0–100 mm). Clinical and laboratory examination was accomplished on the same day as the imaging (FOI; US) examinations. Usually, FOI was ordinarily performed after the US examination. The laboratory investigation included the assessment of erythrocyte sedimentation rate (ESR) and C-reactive protein (CRP). The rheumatoid factor (RF) and anti-citrullinated peptide antibodies (ACPA) were determined at inclusion.

The Disease Activity Score DAS28 was calculated based on the 28-TJC, 28-SJC, ESR or CRP, and patient’s global VAS [1]. The calculation of the difference between DAS28 value at baseline or the prior visit and current value gave information of response to therapy after EULAR response criteria [32–34] (a definition is presented in Additional file 1: Table S1). Based on the EULAR response (Table S1), patients were assigned to a group of responders (DAS28(ESR) ≤ 3.2 and improvement of > 0.6) or non-responders (DAS28(ESR) > 3.2). This process was done to evaluate treatment monitoring by FOI.

### Fluorescence optical imaging (FOI)

FOI (Xiralite® System) was performed following a standardized procedure.

The examination term lasted 6 min, recording one image per second and adding up to a cluster of 360 images [21, 22]. A bolus of indocyanine green (ICG) as fluorescence optical dye with a dose of 0.1 mg/kg body weight was injected intravenously 10 s after the beginning of the examination [21, 22].

The attached software system enabled a visualization of invasion and distribution of ICG in the hands. An image sequence in the film modus and an automatically generated image in the PrimaVistaMode (PVM) were analyzed to evaluate the distribution and enhancement of ICG. For the film modus, three phases in position to the fingertips were defined regarding signal intensities depending on individual perfusion [22]. Phase 1 (p1) included the period between starting the investigation, application of the dye, and increased signal intensities in the fingertips [22], which means an increasing intensity of fluorescence signal. The time period of persisting high signal intensities as plateau in the fingertips was defined as phase 2 (p2) [22]. Investigators do identify this phase on red color signs of the enhancement in the fingertips. The time point without signal intensity meaning only yellow sparkles in the fingertips as a signal for clearance determined the beginning of phase 3 (p3) [22].

Enhancement of ICG can be graded by false-color illustration, which is identical between different scans, time points, and patients. It defines white enhancement as high intensities and concentration of ICG. Red, yellow, and green enhancement follows in a descending order of ICG concentration. For analyzing the joint activity by FOI, the evaluation at the joint level included a combination of size, shape, and color of the signal in a semiquantitative grading system (FOIAS; fluorescence optical imaging activity score): 0 = no signal enhancement, green to yellow signals; 1 = low signal intensity (≤ 25% of the joint area affected), yellow-red signals including red signals with yellow spots; 2 = moderate signal intensity (> 25%, ≤ 50% of the joint area affected), strong red signals including red signals with white spots; 3 = strong signal intensity (> 50% of the joint area affected), white signals [21, 22]. If there was a discrepancy between the intensity of the color and the size of the enhancement, the lower grade of the scoring system was assigned. In detail, enhancements with a discrepancy between two subsequent grades (1 and 2 or 2 and 3) were evaluated with the lower grade number. Differences of signal color and size between grades far apart (e.g., grades 1 and 3) were scored with the intermediate grade (e.g., grade 2).

The ICG distribution in the three phases (p1, p2, p3) and in PrimaVistaMode (PVM) was assessed for the joint regions of 30 joints per patient, including the bilateral wrist, metacarpophalangeal joints (MCP) I–V, proximal interphalangeal joints (PIP) II-V, distal interphalangeal joints (DIP) II-V, and interphalangeal joint of the thumb (IP) [21]. The scoring of color intensity, size, and shape of ICG enhancement was performed by an agreement-based consensus of two investigators (SO; LS).

We calculated the number of affected joints and sum scores (FOIAS; fluorescence optical imaging activity score) for each phase (0–90 scales). In addition, the sum scores of the left hand and the right hand were individually calculated.

### Statistical analyses

Wilcoxon signed rank tests were done to compare clinical data (TJC, SJC, DAS28(ESR)) and FOIAS between two visits (baseline (V0) and 12 months (V12)). Furthermore, Mann-Whitney *U* tests were performed to test for the statistical significance of the difference of score change between responders and non-responders to DAS28 changes under treatment. In addition, we examined whether the FOIAS was correlated with clinical outcome and ultrasound data by use of Spearman’s correlation coefficients including the analysis assessing specific points of time and the change between two points of time. The significance level of 0.05 (5%) was used. *p* values were not adjusted for multiple testing due to the explorative character of the analyses. Statistical analyses were performed with the statistical program R [35]. If not specified otherwise, the descriptive statistics provided median values (1. quartile; 3. quartile).

## Results

Patients’ characteristics are presented in Table 1. Our analyses focused on the 35 of 42 patients who completed the study after 12 months.

**Table 1:** Patients’ characteristics at baseline

		Summary statistics
Age (*n* = 35)	At the beginning of the study	53.32 (13.63) (22.23;74)
	Number of patients ≥ 65 years (LORA)	8/35 (22.86%)
Gender (*n* = 35)	Female	24/35 (68.57%)
Disease duration (*n* = 35)	Duration from initial diagnosis until inclusion in the study (in months)	0.2 (0.42) (0;1.98)
	Duration of symptoms (in months)	1.3 (1.25) (0.13;4.5)
Disease Activity Score 28 (DAS28)	DAS28(ESR) (*n* = 34)	5.55 (1.11) (3.55;7.57)
	DAS28(CRP) (*n* = 31)	5.02 (1.12) (3.09;6.77)
Laboratory parameters	ESR (1 h/mm) (*n* = 34)	39.62 (22.23) (8;95)
	CRP (*n* < 5.0 mg/l) (*n* = 32)	16.34 (17.89) (0;66.52)
Clinical examination (*n* = 35)	Swollen joint count (28-SJC)	6.31 (4.73) (1;20)
	Tender joint count (28-TJC)	10.26 (7.85) (1;25)
VAS (0–100 mm) for disease activity	Patient (*n* = 35)	58.14 (18.79) (20;100)
	Physician (*n* = 21)	54.52 (16.58) (30;90)
Rheumatoid factor (RF)	Seropositive (*n* = 34)	15/35 (42.86%)
	Rheumatoid factor IgA (*n* = 29)	93.23 (166.03) (0.1;500)
	Rheumatoid factor IgM (*n* = 31)	59.64 (113.93) (0.1;500)
Anti-citrullinated peptide antibodies (ACPA) (*n* = 35)	Level	250.34 (393.27) (0.42;1000)
	Positive	4/35 (11.43%)
	Highly positive	15/35 (42.86%)

Patients’ characteristics at baseline: mean (SD); (min; max) or *n* (%); *DAS28* Disease Activity Score of 28 joints, *ESR* erythrocyte sedimentation rate, *CRP* C-reactive protein

### Clinical parameters

During 12 months follow-up, we found statistically significant reductions in TJC 7.0 (3.5;15) to 1.0 (0;3) and SJC 5.0 (3;9.5) to 1.0 (0;2), respectively (each *p* <  0.001; see Table 2).

**Table 2:** Clinical and ultrasound parameters at baseline and after 12 months in total population, group of responders and non-responders

	Month of visit 0 (V0)*	Month of visit 12 (V12)*	Difference between V12 and V0*	*p* value (Wilcoxon signed rank test)
Total population (*n* = 35 for clinical, *n* = 34 for US7 parameters)				
28-SJC	5 (3;9.5) (1;20)	1 (0;2) (0;10)	−4 (−7;− 1) (−19;3)	< 0.001 (sig.)
28-TJC	7 (3.5;15) (1;25)	1 (0;3) (0;26)	−4 (− 10;− 1) (−24;6)	< 0.001 (sig.)
DAS28(ESR)	5.61 (4.8;6.23) (3.55;7.57)	3.31 (2.45;3.98) (1.13;6.19)	−2.22 (−3.11;− 1.39) (−5.45;0.56)	< 0.001 (sig.)
GS-synovitis	7 (5.5;9.5) (2;15)	6 (4.25;7) (2;10)	− 1 (−3;0) (−9;7)	0.0112 (sig.)
GS-tenosynovitis	3 (2;4) (0;5)	2.5 (2;3) (0;4)	−0.5 (−1.75;0) (−4;2)	0.0239 (sig.)
PD-synovitis	4 (3;7) (2;15)	3 (2;3.75) (0;11)	−1 (−3;0) (−9;1)	0.00004 (sig.)
PD-tenosynovitis	3 (2;4) (0;7)	2 (1.25;3) (0;5)	0 (−2;0.75) (−4;1)	0.0163 (sig.)
Group of responders (*n* = 16)				
28-SJC	3.5 (1.75;8.25) (1;15)	0 (0;1) (0;3)	−3.5 (−8.25;− 1) (−14;1)	0.002 (sig.)
28-TJC	4.5 (3;10.5) (1;24)	0 (0;0.25) (0;2)	−3.5 (−10.5;− 3) (−24;− 1)	< 0.001 (sig.)
DAS28(ESR)	4.88 (4.33;6.18) (3.55;7.33)	2.38 (1.89;2.68) (1.13;3.18)	−2.82 (−3.82;− 1.82) (−5.45;− 1.14)	< 0.001 (sig.)
GS-synovitis	8 (6;9.25) (2;12)	6 (4;7) (3;9)	−2 (−4.25;0) (−9;7)	0.038 (sig.)
GS-tenosynovitis	4 (2.75;5) (1;5)	2.5 (2;3) (1;4)	−1 (−2;0) (−4;2)	0.0274 (sig.)
PD-synovitis	4 (3.75;6.5) (2;11)	3 (2;3) (1;4)	−2 (−5;− 0.75) (−8;1)	0.0028 (sig.)
PD-tenosynovitis	3 (2;5) (1;7)	2 (2;3) (1;3)	−1 (−2.25;0) (−4;1)	0.01 (sig.)
Group of non-responders (*n* = 19 for clinical, *n* = 18 for US7 parameters)				
28-SJC	6 (3.5;9.5) (1;20)	1 (0;4) (0;10)	−5 (−6.5;− 2) (−19;3)	0.001 (sig.)
28-TJC	11 (5.5;18.5) (1;25)	3 (1.5;11) (0;26)	−4 (−8;0.5) (−22;6)	0.01 (sig.)
DAS28(ESR)	5.82 (5.34;6.23) (3.61;7.57)	3.95 (3.51;5.21) (3.28;6.19)	−1.62 (−2.61;− 0.51) (−3.73;0.56)	< 0.001 (sig.)
GS-synovitis	7 (4.5;9.5) (2;15)	6 (5;8.5) (2;10)	−1 (−2;0.75) (−5;6)	0.1641
GS-tenosynovitis	2 (2;4) (0;5)	2.5 (1;3) (0;4)	0 (−1;1) (−3;2)	0.506
PD-synovitis	4 (2.5;7) (2;15)	3 (1;5) (0;11)	−1 (−2;0) (−9;1)	0.0058 (sig.)
PD-tenosynovitis	2 (1;3) (0;7)	2.5 (1;3) (0;5)	0 (−0.75;1) (−4;1)	0.7732

Clinical and ultrasound (US) parameters at baseline and after 12 months in total population, group of responders and non-responders: *Median (1. quartile; 3. quartile); (min; max), significance level = 0.05; *28-SJC* swollen joint count of 28 joints, *28-TJC* tender joint count of 28 joints, *DAS28(ESR)* Disease Activity Score of 28 joints and erythrocyte sedimentation rate (ESR), *GS* greyscale mode in ultrasound, *PD* Power Doppler mode in ultrasound

At baseline, patients had high disease activity with median DAS28(ESR) of 5.61 (4.8;6.23). After 1 year, disease activity was statistically significantly reduced to a median DAS28 of 3.31 (2.45;3.98) (*p* <  0.001) which corresponds to moderate disease activity (see Table 2). By the end of the study, 31.4% (11/35) of patients had achieved remission (DAS28 < 2.6).

### Fluorescence optical imaging (FOI)

**Fig. 1:**
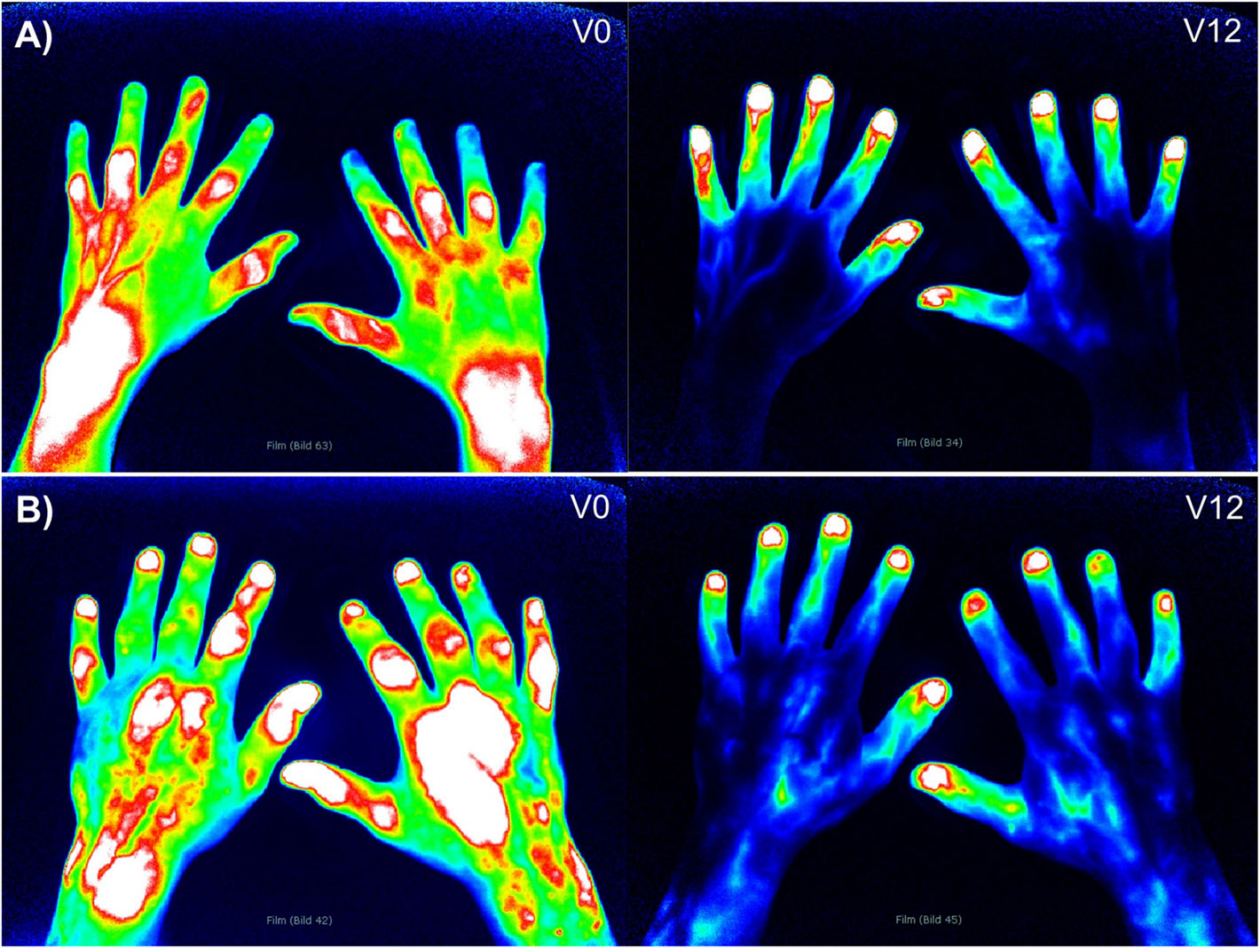
Reduction of early enhancement in FOI (fluorescence optical imaging) phase 1 after 12 months follow-up: **a** V0: Example with early high enhancement in phase 1 before ICG flooding in the fingertips, especially in the wrists, PIPs, and IPs of both hands. Moderate enhancement in MCP II and IV of the right hand. V12: High physiological enhancement in the fingertips in phase 1 after 12 months. No enhancement in the finger and hand joints. **b** Example of early enhancement in phase 1 in both hands, especially in MCP II and III of the right hand. High enhancement also in PIPs of both hands, left wrist, and MCP II and III. Physiological signal in the fingertips. V12: High physiological enhancement in the fingertips in phase 1 after 12 months. No significant enhancement in the finger and hand joints. V0: baseline, V12: follow-up after 12 months

Statistically significant reductions were detected in the FOI sum score (FOIAS) in phase 1 from baseline (5.0, (1.04; 26)) to 12 months follow-up (1.0 (0; 4)) in the total patient cohort (*p* = 0.0045). There were no statistically significant changes in the FOI sum score in phase 2, phase 3, or PVM in the total cohort (see Table 3 and Fig. 1 and Additional file 1: Table S2 (analysis without DIP).

**Table 3:** FOIAS of phases 1–3 and PVM (PrimaVistaMode) in FOI at baseline and after 12 months (*n* = 35 for FOIAS parameters

	Month of visit 0 (V0)*	Month of visit 12 (V12)*	Difference between V12 and V0*	*p* value (Wilcoxon signed rank test)
Phase 1	5 (1.04;26) (0;70.91)	1 (0;4) (0;32)	−3 (− 17;0) (−69.91;12)	0.00445 (sig.)
Phase 2	16 (10.5;25) (1;40)	16 (9.5;24) (2;43)	2 (−4.5;6.81) (− 18;18)	0.6004
Phase 3	1 (0;2.5) (0;15)	1 (0;4) (0;10)	0 (− 1;1.5) (− 12;9)	0.5451
PVM	9 (4.5;13) (0;24)	9 (3.5;14) (0;26)	−1 (−4;4) (− 15;14)	0.7461

Phases 1–3 and PVM (PrimaVistaMode) of FOI at baseline and after 12 months (*n* = 35): *FOIAS* fluorescence optical imaging activity score; *median (1. quartile; 3. quartile); (min; max); significance level = 0.05

### Correlation of FOI and clinical parameters

**Fig. 2:**
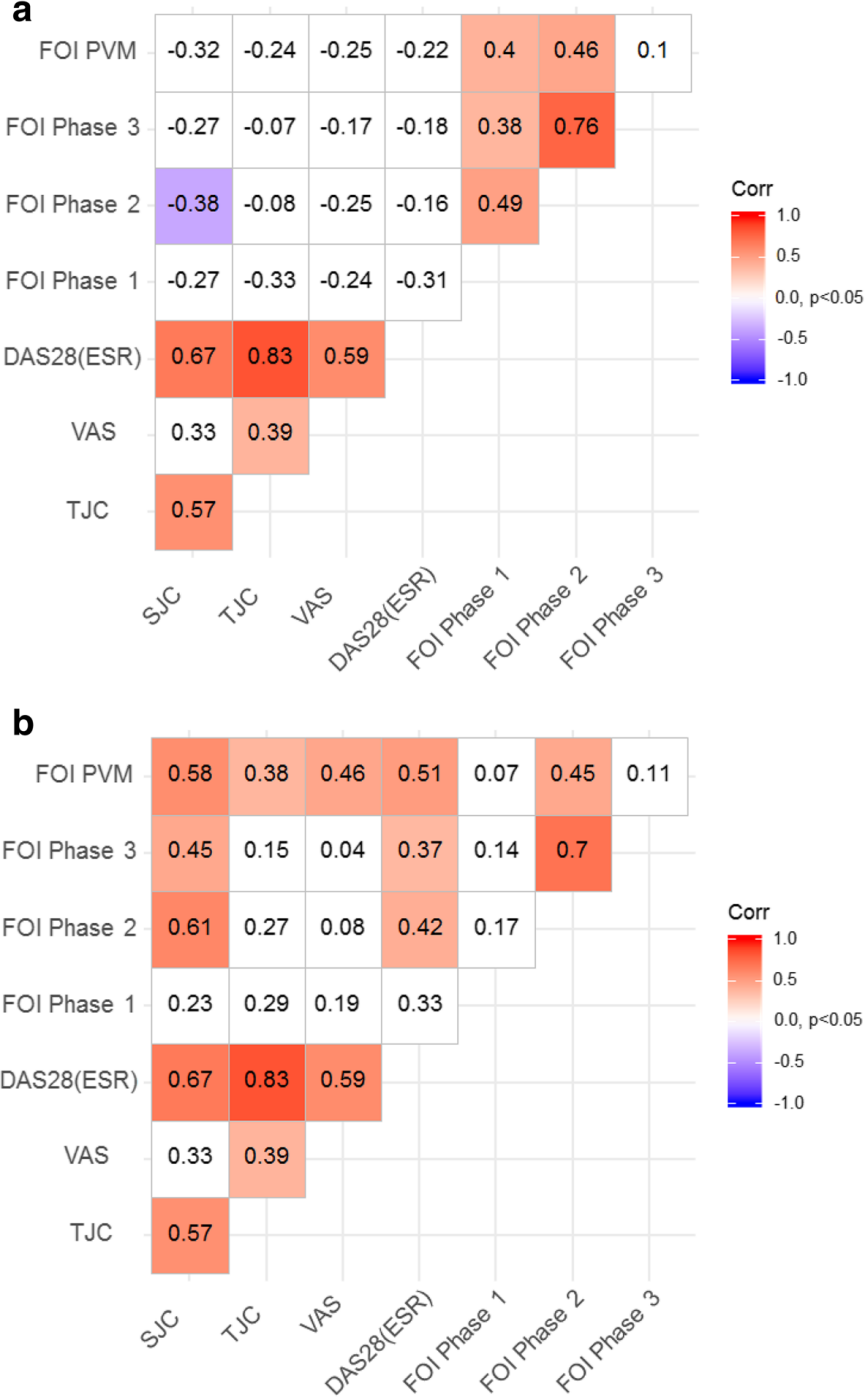
Pairwise correlation between the number of affected joints in clinical examination and FOI phases 1, 2, and 3 and PVM: significance level = 0.05; FOI = fluorescence optical imaging; PVM = PrimaVistaMode; SJC = swollen joint count; TJC = tender joint count; DAS28(ESR) = Disease Activity Score of 28 joints and erythrocyte sedimentation rate (ESR); VAS = visual analog scale. Spearman’s correlation coefficients and *p* values are presented. **a** measurement at V0 (baseline) **b** change at V12 (12 months follow-up)

Regarding baseline data, no significant positive correlation between FOI and clinical parameters (TJC, SJC, and DAS28(ESR)) can be shown (see Fig. 2a).

FOI in phase 1 showed no statistically significant correlations with clinical data concerning the analysis of clinically affected joints and FOI after 12 months follow-up (see Fig. 2b). FOI phases 2 and 3 demonstrated weak to moderate correlations with DAS28(ESR) and SJC. PVM correlated significantly with all three clinical parameters. While the correlation with TJC was only weak (*r* = 0.38), PVM correlated moderately with SJC and DAS28(ESR) (*r* = 0.58, *r* = 0.51, see Fig. 2b). The corresponding *p* values of the correlation coefficients were < 0.05. An additional statistical analysis performed without the DIP joints in FOI did not change the level or direction of correlation (for further data see Additional file 1: Tables S3a-b).

### Correlations between FOI and US

US data of baseline and after 12 months are presented in Table 2.

**Fig. 3:**
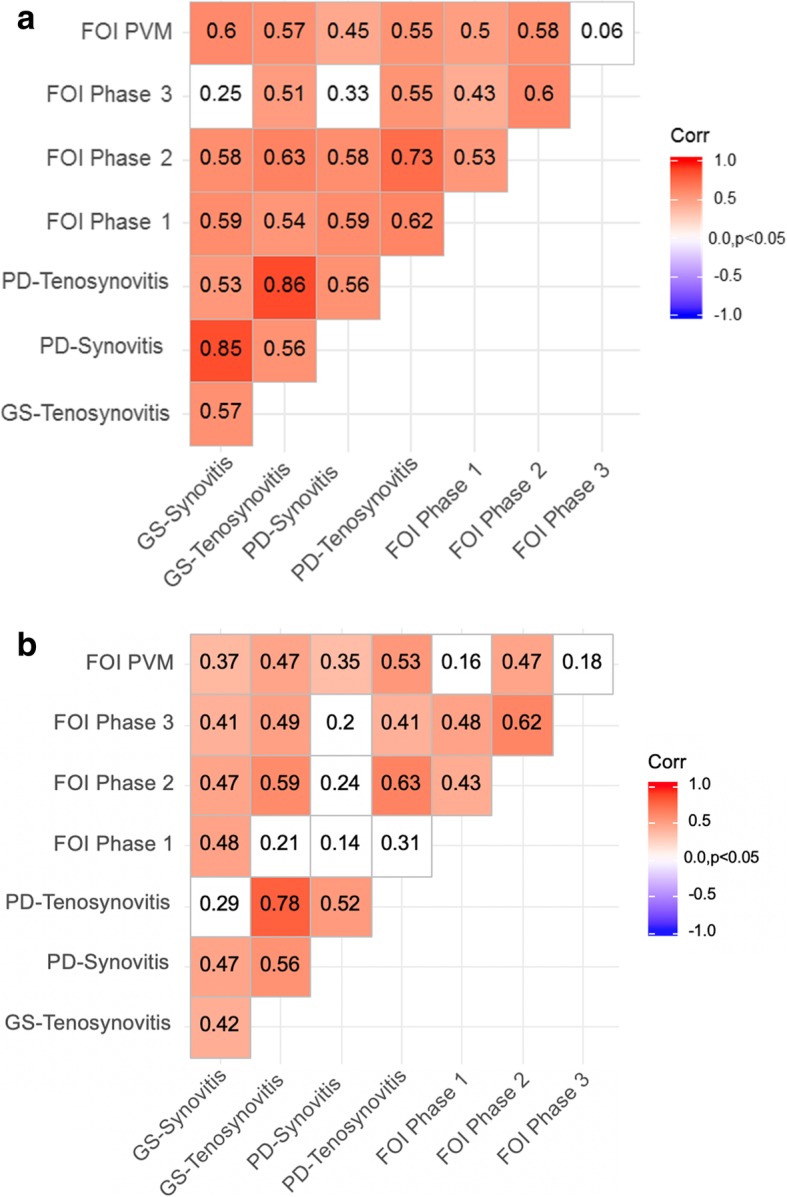
Pairwise correlation between US7 and FOI phase 1, 2, and 3 and PVM: significance level = 0.05; FOI = fluorescence optical imaging; PVM = PrimaVistaMode; PD = power Doppler mode in ultrasound, GS = greyscale mode in ultrasound. Spearman’s correlation coefficients and *p* values are presented. **a** measurements at V0 (baseline) **b** change at V12 (12 months follow-up)

With respect to the calculated correlations in the total group, tenosynovitis in greyscale ultrasound (GSUS) and PDUS correlated with every phase and PVM at baseline, but strongest for PD-tenosynovitis with phase 2 (*r* = 0.73; *p* <  0.05). With regard to GS- and PD-synovitis, significant positive correlations with phase 1, phase 2, and PVM at baseline can be shown. While GS-synovitis most strongly correlated with PVM in FOI (*r* = 0.6), the strongest correlation between PD-synovitis and FOI was demonstrated for phase 1 (*r* = 0.59) (Fig. 3a).

After 12 months, FOI in phase 2 demonstrated the strongest correlations, especially with PD-tenosynovitis (*r* = 0.63; *p* <  0.05) and GS-tenosynovitis (*r* = 0.59; *p* <  0.05). For phase 1, low correlation with GS-synovitis can be demonstrated (*r* = 0.48, *p* <  0.05). However, PVM was the only FOI part correlating with all considered US parameters (Fig. 3b).

### Comparison of clinical findings and FOI in responders vs. non-responders

According to the EULAR response criteria [32], *n* = 16 (45.7%) patients were defined as responders (DAS28(ESR) ≤ 3.2 and improvement of > 0.6) and *n* = 19 (54.3%) as non-responders (DAS28(ESR) > 3.2).

Regarding the responders (DAS28(ESR) ≤ 3.2 and improvement of > 0.6; *n* = 16) and non-responders (DAS28(ESR) > 3.2; *n* = 19), both groups demonstrated a statistically significant decline in affected tender and swollen joints after 12 months. Non-responders had a greater number of tender (11 (5.5;18.5)) and swollen (6; (3.5;9.5)) joints at baseline than the group of responders (TJC: 4.5 (3;10.5) and SJC: 3.5 (1.75;8.25), respectively). After 12 months, the number of tender and swollen joints decreased to 0 in responders (see Table 2). In non-responders, the median number of tender joints and swollen joints was 3 (1.5;11) and 1 (0;4), respectively (see Table 2).

With regard to US7 score parameters, the group of responders showed significant decrease in all US7 parameters, while the group of non-responders revealed significant decrease only in PD-synovitis.

**Fig. 4:**
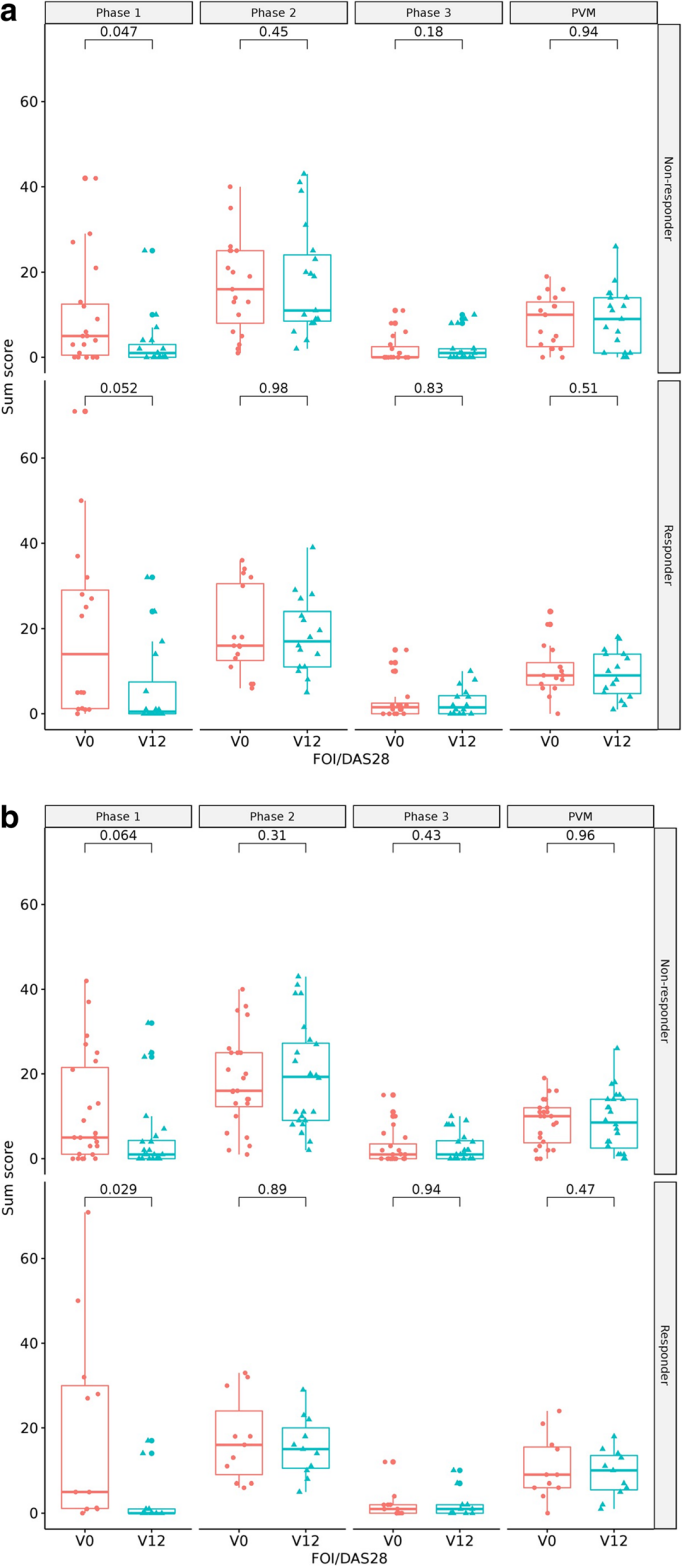
**a** Course of FOI sum scores in comparison to EULAR response criteria of “low disease activity” according to DAS28. **b** Course of FOI sum scores in comparison to EULAR response criteria of “remission” according to DAS28: non-responder (DAS28(ESR) > 3.2), responder (DAS28(ESR) ≤3.2 and improvement of > 0.6). FOI = fluorescence optical imaging; DAS28 = Disease Activity Score of 28 joints. V0 (red): baseline, V12 (blue): follow-up after 12 months

We found no statistically significant difference in the change of the FOI sum score between responders and non-responders for the FOI phases 2 and 3 and PVM relating to EULAR response criteria [32–34] for low disease activity. In FOI phase 1, the difference in the change of FOI sum score was similar in non-responders (*p* = 0.047) and responders (*p* = 0.052) (see Fig. 4a). There were no statistically significant differences across patients who did vs. did not achieve remission for FOI phases 2 and 3 and PVM. For FOI phase 1, statistically significant difference was shown only in responders regarding clinical remission status (see Fig. 4b).

## Discussion

To our knowledge, this present study is the first one describing the changes of FOI in a homogeneous cohort of patients with early and active RA over a period of 12 months under antirheumatic therapy.

Regarding clinical and laboratory disease activity, DAS28 decreased from high disease activity (DAS28 = 5.61) to moderate (DAS28 = 3.31) over the time period described. 31.4% of patients (*n* = 16) achieved remission of DAS28 < 2.6 under antirheumatic therapy.

Concerning FOI, we found significant reductions in the FOI sum score in phase 1 in the total cohort, whereas the other phases remained stable. These results are in line with previous results by Meier et al., who found a significant reduction in early signal intensity after 24 weeks of therapy using a computer-based evaluation of FOI [30]. In a study by Werner et al., phase 1 featured the highest agreement between clinical examination and FOI. In addition, the highest specificity was calculated for phase 1 compared to MRI and US in this study [21], which was also confirmed by Krohn et al. [24]. Besides, phase 1 did not show any pathological changes in a healthy cohort, confirming the hypothesis that this early phase reflects active inflammation with increased vascularization and therefore high clinical disease activity [21]. In a previous study, we examined FOI in patients with either osteoarthritis (OA) or RA. OA patients showed significantly less activity in phase 1 (maximum degree 1), but a consistently high signal accumulation in phase 2 (especially in the wrist) [26]. These results support the hypothesis that phase 1 detects an active inflammation [21, 22] since OA is usually characterized by a less pronounced inflammation as compared to systemic inflammatory joint diseases.

In our analysis, we did not find a significant correlation between the change of phase 1 and joint count. The lack of correlations between phase 1 and clinical parameters may be due to different parameters we assess in clinical examination and FOI. While we investigate a disturbed microcirculation in FOI, we document morphological changes in swollen fingers as a result of infiltrated cells in the synovial membrane and pannus. The impaired microcirculation visualized by FOI comprises neoangiogenesis, hyperperfusion, and capillary leakage within the inflammatory process of RA [36]. It may be assumed that both pathologies (microcirculation and morphological changes) within the articular region appear on different time points in the disease course. On the other hand, neoangiogenesis is an important component in the formation of pannus and, therefore, not only found around the inflamed joint [36]. Another explanation for the lack of correlation may be false-positive findings of FOI. However, Werner et al. demonstrated a low rate of false-positive findings between 0.5 and 5% by FOI [22]. Thus, our results stand in contrast to the results by Werner et al. who presented good agreement rates and low, but significant, correlations between FOI and clinical examination. It should be noted that FOI detects any inflammation including scratches, plaques, and insect bites. On this account, the evaluation requires a well-trained investigator [22]. However, the localization, form, and temporal distribution of enhancement in the individual FOI phases allow a differentiation [22] and may indicate the underlying pathology or possible disease [26].

In addition, Werner et al. suggested divergence between local inflammation in the hand and systemic inflammation [21]. Furthermore, high variance for components of the DAS28 was recently described [37], while FOI is a more objective technical tool.

After 1 year under csDMARD or bDMARD therapy, approximately 46% of the patients in our study achieved a clinical response stage according to the EULAR response criteria [32–34] and showed a reduction in disease activity according to the treat-to-target principle (T2T). The change in FOI sum scores was similar in patients with and without a clinical response. The significant reductions in phase 1 also in non-responders probably show a more objective reduction of inflammation by FOI, while the clinical non-response can also depend on individual person-related (i.e., psychological) factors which may lead to elevated global disease activity on VAS. Recently, Hammer et al. showed no association between tender joints and synovitis in GSUS and PDUS, while a strong correlation between swollen joints and US synovitis was calculated. In addition, they found a primary associationn between tender joints and patient-reported joint pain [38]. These findings support that the parameters of DAS28 hardly correlated with the sum scores of FOI also in this present study. Similar results for FOI were published by Werner et al. assuming different characteristics of pathologies [21].

We conducted correlation analyses between musculoskeletal ultrasound and FOI findings. At baseline examination, strong correlations were found between all FOI phases and the ultrasound parameters, especially tenosynovitis in PDUS. The strongest correlations were found for FOI phase 2 at both baseline and after 12 months of therapy. In contrast, phase 1 did only show a positive correlation with synovitis in GSUS after 12 months follow-up. The meaning of phase 2 as a marker of subclinical activity has already been discussed in previous studies [21, 22, 24]; however, no longitudinal data exist yet showing a predictive value of phase 2, for example in terms of erosive disease or flare prediction. The strong signal accumulation could be caused by increased vascularization due to chronic inflammation. The low correlations of phase 1 with US at month 12 may indicate that there was no or only a small amount of acute inflammation after 1 year of intensive therapy, whereas the greyscale US synovitis findings can persist. FOI phase 1 is probably a reflection of acute inflammation. A good response of early enhancement in FOI to therapeutic interventions demonstrated by Meier et al. showed a decrease of early signal intensity after 6 months in response to therapy [30].

## Conclusions

In conclusion, activity in FOI phase 1 changed significantly over 1 year under therapy in the group of responders regarding the parameter of clinical remission (DAS28). However, a significant change of FOI phase 1 was also observed in non-responders, so we cannot objectively deduce a therapy response of phase 1. However, the correlation of FOI with ultrasound as a validated and well-established imaging technique in daily rheumatological practice should be emphasized. In the issue, the role of FOI in therapy monitoring needs to be investigated in further studies.

## Supplementary information

**Additional file 1.** The supplementary material gives further information on methods including patient consent, EULAR response criteria and ultrasound. It reveals additional results of FOIAS in phases 1-3 and PVM (PrimaVistaMode) of FOI at baseline and after 12 months as well as correlation to number of clinically affected joints with DIP joint excluded in the analyses. (DOCX 39 kb)

## Data Availability

The datasets used and/or analyzed during the current study are available from the corresponding author on reasonable request.

## Abbreviations

ACPA: Anti-citrullinated peptide antibodies
bDMARDs: Biologic disease-modifying antirheumatic drugs
BMBF: Federal Ministry of Education and Research (Bundesministerium für Bildung und Forschung)
csDMARDs: Conventional synthetic disease-modifying antirheumatic drugs
CRP: C-reactive protein
DAS28: Disease Activity Score of 28 joints
DIP: Distal interphalangeal joints
ESR: Erythrocyte sedimentation rate
EULAR: European League Against Rheumatism
FOI: Fluorescence optical imaging
FOIAS: Fluorescence optical imaging activity score
GSUS: Greyscale ultrasound
ICG: Indocyanine green
IP: Interphalangeal joint of the thumb
MCP: Metacarpophalangeal joints
MRI: Magnetic resonance imaging
OA: Osteoarthritis
PDUS: Power Doppler mode, power Doppler ultrasound
PIP: Proximal interphalangeal joints
PVM: PrimaVistaMode
p1: Phase 1 in FOI
p2: Phase 2 in FOI
p3: Phase 3 in FOI
RA: Rheumatoid arthritis
RF: Rheumatoid factor
SJC: Swollen joint count
TJC: Tender joint count
T2T: Treat-to-target principle
US: Musculoskeletal ultrasound, ultrasonography
VAS: Visual analog scale
V0: Baseline visit
V12: Visit after 12 months
