# Supplementary material for: Fluorescence optical imaging for treatment monitoring in patients with early and active rheumatoid arthritis in a 1-year follow-up period
Source: Arthritis Res Ther. 2019 Sep 18;21:209. doi: 10.1186/s13075-019-1989-5 (PMC6749637; doi:10.1186/s13075-019-1989-5)
Supplement: Supplementary file 1 — Additional file 1. The supplementary material gives further information on methods including patient consent, EULAR response criteria and ultrasound. It reveals additional results of FOIAS in phases 1-3 and PVM (PrimaVistaMode) of FOI at baseline and after 12 months as well as correlation to number of clinically affected joints with DIP joint excluded in the analyses. (DOCX 39 kb) [file 13075_2019_1989_MOESM1_ESM.docx]

**Additional file 1**

**Patients and Methods**

The approval of the study was given by the local ethics committee of Charité - Universitätsmedizin Berlin (EA1/193/10). After detailed information in written and oral form, all included patients gave consent to participate in the study. All patients were older than 18 years and enrolled at the outpatient clinic of the department of Rheumatology, Charité - Universitätsmedizin, Berlin; Germany.

**Clinical and laboratory examination**

Clinical examination was performed at each patient’s visit. Duration of morning stiffness and treatment tolerability were interrogated. Concerning laboratory examination, liver and kidney parameters were also documented for estimation of therapy tolerability.

Referring to the EULAR response criteria the status of remission was assessed (see also **Table S1**) (1-3):

- DAS28 < 2.6: clinical remission
- DAS28 ≥ 2.6 to ≤ 3.2: low disease activity
- DAS28 > 3.2 to ≤ 5.1: moderate disease activity
- DAS28 > 5.1: high disease activity.

**Musculoskeletal ultrasound**

The same rheumatologist who did the clinical examination performed an additional ultrasound examination of the clinically dominant (for tenderness and swelling) hand and forefoot using the US7 score (4).

The US7 score includes the evaluation of synovitis, tenosynovitis and erosion of seven selected joints (wrist, metacarpophalangeal joint (MCP) II + III, proximal interphalangeal joint (PIP) II + III, metatarsophalangeal joint (MTP II + V). US included two different modes: Greyscale Ultrasound (GSUS = Brightness scan) and power Doppler US (PDUS). In addition, the investigation of the respective joints was performed in different planes: dorsomedian (midline), palmomedian and ulnar for the wrist, palmar and dorsal for the finger joints. The toe joints were dorsally examined for synovitis. Erosions were investigated on palmar/plantar and dorsal side as well as radial on MCP 2 and lateral on MTP 5. The evaluation of synovitis in GSUS as well as synovitis and tenosynovitis in PDUS was realized by a semi quantitative score (0-3). Tenosynovitis in GSUS as well as erosions were graded by a qualitative score in dichotomous manner (0/1). Finally, the sum of the synovitis score in GSUS (0-27) and PDUS (0-39), the sum of the tenosynovitis score (0-7) and PDUS (0-21) as well as the erosions (0-14) of the US7 score were calculated (4).

In order to reach comparability of US and FOI, the joints of the foot were excluded from our analyses, and a sum score was built of the five joints of the investigated hand. For the single joint analysis, only the highest value of different regions of a single joint was used. That refers to the dorsomedian, ulnar or palmomedian region in the wrist and dorsal or palmar region in the fingers joints. The sum of the respective values resulted in a 5-joint sum score in US. In addition, on the basis of the individual scores, the joint planes could also be viewed and compared separately with the FOI.

**Table S1:** EULAR response criteria by DAS28 (1-3)

| **current DAS28** | **difference of DAS28 to prior value** | | |
| --- | --- | --- | --- |
|  | **> 1,2** | **> 0,6 und ≤ 1,2** | **≤ 0,6** |
| **≤ 3,2** | ***good response*** |  |  |
| **> 3,2 und ≤ 5,1** |  | ***moderate response*** |  |
| **>5,1** |  |  | ***no response*** |

**Table S2:** FOIAS of phases 1-3 and PVM (PrimaVista mode) of FOI at baseline and after 12 months – DIPs excluded (n=35)

|  | **Month of visit 0 (V0)*** | **Month of visit 12 (V12)*** | **Difference between V12 and V0*** | **P-value (Wilconxon signed rank test)** |
| --- | --- | --- | --- | --- |
| **Phase 1** | 4.55(0.91;22.27)  (0;47.27) | 0.91(0;2.73)  (0;24.55) | -1.82(-14.09;0)  (-46.36;10.91) | 0.0022 (sig.) |
| **Phase 2** | 12.73(7.73;20.91)  (0.91;33.64) | 11.82(7.73;19.55)  (1.82;31.82) | 1.82(-3.18;4.09)  (-14.55;15.45) | 0.7005 |
| **Phase 3** | 0.91(0;2.27)  (0;12.73) | 0.91(0;3.64)  (0;9.09) | 0(-0.91;1.36)  (-10.91;7.27) | 0.5398 |
| **PVM** | 7.27(3.18;10)  (0;18.18) | 7.27(2.73;10.91)  (0;15.45) | -0.91(-3.64;2.73)  (-13.64;8.18) | 0.5919 |

**Table S2:** FOIAS of phases 1-3 and PVM (PrimaVista mode) of FOI at baseline and after 12 months – DIPs excluded (n=35): Median (1. Quartile; 3. Quartile); (Min; Max); Significance level = 0.05; PVM = PrimaVistaMode

**Table S3a:** Pairwise correlation between the number of affected joints in clinically examination and FOI Phase 1, 2, 3 and PVM (DIPs excluded)– measurements at V0

| **Spearman’s correlation coefficient** | **SJC** | **TJC** | **DAS28(ESR)** |
| --- | --- | --- | --- |
| **FOI Phase1** | -0.29 | -0.34 | -0.32 |
| **FOI Phase 2** | -0.45 (sig.) | -0.19 | -0.27 |
| **FOI Phase 3** | -0.28 | -0.08 | -0.19 |
| **FOI PVM** | -0.44 (sig.) | -0.35 (sig.) | -0.33 |

**Table S3a:** Pairwise correlation between the number of affected joints in clinically examination and FOI Phase 1, 2, 3 and PVM – measurements at V0: Significance level = 0.05; SJC = swollen joint count, TJC = tender joint count, DAS28 (ESR) = Disease Activity Score of 28 joints and erythrocyte sedimemtation rate (ESR)

**Table S3b:** Pairwise correlation between the number of affected joints in clinically examination and FOI Phase 1, 2, 3 and PVM (DIPs excluded)– change at V12

| **Spearman’s correlation coefficient** | **SJC** | **TJC** | **DAS28(ESR)** |
| --- | --- | --- | --- |
| **FOI Phase1** | 0.2 | 0.27 | 0.3 |
| **FOI Phase 2** | 0.6 (sig.) | 0.25 | 0.45 (sig.) |
| **FOI Phase 3** | 0.47 (sig.) | 0.2 | 0.42 (sig.) |
| **FOI PVM** | 0.59 (sig.) | 0.36 (sig.) | 0.48 (sig.) |

**Table S3b:** Pairwise correlation between the number of affected joints in clinically examination and FOI Phase 1, 2, 3 and PVM – change at V12: Significance level = 0.05; SJC = swollen joint count, TJC = tender joint count, DAS28 (ESR) = Disease Activity Score of 28 joints and erythrocyte sedimemtation rate (ESR)

**References for supplementary material**

1. van Gestel AM, Prevoo ML, van 't Hof MA, van Rijswijk MH, van de Putte LB, van Riel PL**.** Development and validation of the European League Against Rheumatism response criteria for rheumatoid arthritis. Comparison with the preliminary American College of Rheumatology and the World Health Organization/International League Against Rheumatism Criteria. Arthritis Rheum. 1996;39(1):34-40.

2. van Gestel AM, Haagsma CJ, van Riel PL**.** Validation of rheumatoid arthritis improvement criteria that include simplified joint counts. Arthritis Rheum. 1998;41(10):1845-50.

3. van Gestel AM, Anderson JJ, van Riel PL, Boers M, Haagsma CJ, Rich B, et al. ACR and EULAR improvement criteria have comparable validity in rheumatoid arthritis trials. American College of Rheumatology European League of Associations for Rheumatology. J Rheumatol. 1999;26(3):705-11.

4. Backhaus M, Ohrndorf S, Kellner H, Strunk J, Backhaus TM, Hartung W, et al. Evaluation of a novel 7-joint ultrasound score in daily rheumatologic practice: a pilot project. Arthritis Rheum. 2009;61(9):1194-201.
